# The glycolytic enzyme ALDOA and the exon junction complex protein RBM8A are regulators of ribosomal biogenesis

**DOI:** 10.3389/fcell.2022.954358

**Published:** 2022-09-14

**Authors:** Jessica Denise Schwarz, Sören Lukassen, Pranjali Bhandare, Lorenz Eing, Marteinn Thor Snaebjörnsson, Yiliam Cruz García, Jan Philipp Kisker, Almut Schulze, Elmar Wolf

**Affiliations:** ^1^ Cancer Systems Biology Group, Theodor Boveri Institute, University of Würzburg, Würzburg, Germany; ^2^ Center for Digital Health, Berlin Institute of Health at Charité—Universitätsmedizin Berlin, Berlin, Germany; ^3^ Tumor Metabolism and Microenvironment, German Cancer Research Center, Heidelberg, Germany

**Keywords:** ribosome biogenesis, Ribosomal protein gene, genetic screen, genome-wide screen, RBM8A, Y14, AldoA, aldolase A

## Abstract

Cellular growth is a fundamental process of life and must be precisely controlled in multicellular organisms. Growth is crucially controlled by the number of functional ribosomes available in cells. The production of new ribosomes depends critically on the activity of RNA polymerase (RNAP) II in addition to the activity of RNAP I and III, which produce ribosomal RNAs. Indeed, the expression of both, ribosomal proteins and proteins required for ribosome assembly (ribosomal biogenesis factors), is considered rate-limiting for ribosome synthesis. Here, we used genetic screening to identify novel transcriptional regulators of cell growth genes by fusing promoters from a ribosomal protein gene (*Rpl18*) and from a ribosomal biogenesis factor (*Fbl*) with fluorescent protein genes (RFP, GFP) as reporters. Subsequently, both reporters were stably integrated into immortalized mouse fibroblasts, which were then transduced with a genome-wide sgRNA-CRISPR knockout library. Subsequently, cells with altered reporter activity were isolated by FACS and the causative sgRNAs were identified. Interestingly, we identified two novel regulators of growth genes. Firstly, the exon junction complex protein RBM8A controls transcript levels of the intronless reporters used here. By acute depletion of RBM8A protein using the auxin degron system combined with the genome-wide analysis of nascent transcription, we showed that RBM8A is an important global regulator of ribosomal protein transcripts. Secondly, we unexpectedly observed that the glycolytic enzyme aldolase A (ALDOA) regulates the expression of ribosomal biogenesis factors. Consistent with published observations that a fraction of this protein is located in the nucleus, this may be a mechanism linking transcription of growth genes to metabolic processes and possibly to metabolite availability.

## Introduction

For multicellular organisms, growth and proliferation of cells is an essential need during development, but also throughout the whole lifespan of an organism. In order to grow, cells need to synthesize proteins, which in turn are made by ribosomes. The amount of ribosomes in a cell is a major determinant of translational output, which is essential for growth (Chaillou et al., 2014). Therefore, much of a cell’s energy is spent on ribosome biogenesis, the process required to build ribosomes. About 7,500 ribosomes are synthesized per minute in a proliferating HeLa cell (Mayer and Grummt, 2006); with 80 ribosomal proteins (RPs) (Gilles et al., 2020), this corresponds to approximately 600,000 RP molecules. During this time, a total of approximately 2 × 10^6^ functional proteins are produced (Yewdell, 2001), indicating that RP production accounts for up to 30% of all protein biosynthesis events (Schwarz, 2022).

However, RPs on their own are not capable of producing functional ribosomes. For this purpose, the action of all three RNA polymerases is required. RNA polymerase I (POL I) transcribes the 47S pre-rRNA precursor, which is further processed into 18S, 5.8S, and 28S rRNA (Cory and Adams, 1977; Kominami et al., 1981; Gonzalez and Sylvester, 1995; Mullineux and Lafontaine, 2012). In mammals, several hundred tandemly repeated ribosomal DNA (rDNA) clusters are transcribed in the nucleolus to generate the 47S pre-rRNA precursor molecules (Henras et al., 2015). RNA polymerase III (POL III) is required for the transcription of 5S rRNA (Sakonju et al., 1980) from multiple gene copies (Fedoriw et al., 2012). rRNA synthesis poses a huge energetic effort to cells since rRNAs are the dominant RNA species, accounting for about 80% of the total amount of RNA in mammalian cells.

RNA polymerase II (POL II) in turn does not only transcribe the genes of the 80 ribosomal proteins present in eukaryotic ribosomes but also more than 200 ribosome biogenesis factors (RiBis) needed for the assembly of ribosomes. Moreover, POL II transcribes non-coding small nucleolar RNAs (snoRNAs) which are involved in the modification and maturation of rRNAs together with RiBis. An example of an important ribosomal biogenesis factor associated with C/D box snoRNAs is the methyltransferase fibrillarin (Yu and Li, 2017), which catalyses the site-specific transfer of a methyl group from *S*-Adenosyl methionine to a ribose 2′-hydroxyl group on its target rRNA.

Because protein production and ribosome biogenesis are of particular importance for growth and proliferation, cells must be able to rapidly adapt ribosome biosynthesis to environmental changes that promote or disfavour growth. Indeed, cells can sense mitogen availability for example *via* the phosphatidylinositol-3-kinase (PI3K)/AKT/mammalian target of rapamycin (mTOR) pathway and adjust ribosome biogenesis and thus cell growth and proliferation accordingly (Iadevaia et al., 2014; Yang et al., 2019). mTOR phosphorylates ribosomal protein S6 kinases (S6Ks) and eukaryotic initiation factor (eIF) 4E-binding proteins (4E-BPs) among others, thereby promoting translation (Valvezan and Manning, 2019).

Growth-promoting pathways acting at the post-transcriptional level such as mTOR, are relatively well understood at a mechanistic level. Literature suggests that mTOR is also involved in the transcriptional regulation of RiBis and/or RPs (Mayer and Grummt, 2006; Chauvin et al., 2014; Rosario et al., 2020). Transcription factors regulating ribosomal biogenesis need to be able to balance the transcriptional outputs of all three RNA polymerases. In yeast, several transcription factors regulating RPs and/or RiBis were discovered (Lempiainen and Shore, 2009). For instance, the zinc-finger protein Sfp1 and the serine/threonine protein kinase Sch9 regulate RiBis and RPs in *Saccharomyces cerevisiae* (Lempiainen and Shore, 2009). In contrast, Fhl1 and Ifh1 are thought to be specific regulators of RPs (Jorgensen et al., 2002; Shore et al., 2021), while Dot6 and Tod6 are involved in RiBi-specific regulation downstream of TORC1 activity in yeast (Lippman and Broach, 2009).

Our understanding of the mechanisms of transcriptional regulation of RP and RiBi genes in mammals is still very limited (Hu and Li, 2007; Petibon et al., 2021). However, genes involved in ribosomal biogenesis contain conserved sequence motifs in their promoters, suggesting their specific regulation by transcription factors. One example is the localized tandem sequence motif (LTSM), localized approximately 60 base pairs downstream of the transcription start site in the first intron of ribosomal protein genes (Roepcke et al., 2011). A number of other motifs are found in the promoters of human RP genes, including SP1, GABP or YY1 binding sites (Perry, 2005). In contrast, some yeast proteins involved in ribosome biogenesis have no obvious mammalian homologue, as for example Ifh1 (Schwarz, 2022). Conversely, many of the human ribosome biogenesis factors do not have a yeast homolog (Tafforeau et al., 2013).

All these observations suggest that mammalian ribosomal biogenesis is indeed regulated at the transcriptional level in mammals but that not all regulators have been identified so far. To gain deeper insight into the transcriptional regulation of ribosomal proteins and ribosome biogenesis factors in mammals, we thus performed a genome-wide CRISPR/Cas9 knockout reporter screen to identify novel regulatory proteins. Surprisingly, our screen identified not only several components of the core transcription machinery, but also the glycolytic enzyme ALDOLASE A (ALDOA) and the exon junction complex protein RBM8A as being essential for the transcription of ribosomal biogenesis genes.

## Materials and Methods

### Materials

Information about the antibodies used in this study can be found in Supplementary Table S1. Information about the amino acid sequences of the fluorescent reporters used in this study can be found in Supplementary Table S2. Information about the oligonucleotides used in this study can be found in Supplementary Table S3 (primers used to amplify the promoters; primers used to amplify the sgRNAs from genomic DNA; sgRNA sequences; qRT-PCR primers; siRNA sequences). Differential expression results of the 4sU-Seq analysis excluding exonic reads are given in Supplementary Table S4. Differential expression results of the 4sU-Seq analysis containing intronic and exonic reads are given in Supplementary Table S5.

## Methods

### Cycloheximide assay

U2OS cells stably expressing SFFV-driven EGFP-PEST or HEK cells stably expressing SFFV-driven tGFP or SFFV-driven tGFP-PESTmut were treated with 10 μg/ml (U2OS) or 100 μg/ml (HEK) CHX for 0 min, 30 min, 1 h, 2 h, 4 h or 8 h and analyzed by FACS. Analysis was performed using FlowJo v10. Log10 of the median fluorescence intensity (% of median fluorescence of the 0 h time point) was plotted.

### Promoter testing by quantitative real-time PCR

T lymphoma cells were treated with 1 μg/ml of Doxycycline (Dox) for 16 h–24 h. RNA extraction was performed using the RNeasy Mini Kit (Qiagen, Hilden) according to manufacturer’s instructions, including on-column DNAse I digestion, followed by cDNA synthesis. qRT-PCR was performed in triplicates. The ΔΔCT method was used to calculate relative expression values. *Actb* was used as a reference gene.

### Screening cell line generation

Murine NIH/3T3 cells were infected with lentiviral *Fbl* promoter-driven EGFP-PEST and *Rpl18* promoter-driven tRFP-PEST constructs, selected with Hygromycin and a single cell clone was generated.

### Genome-wide CRISPR/Cas9 knockout screen

100 ng DNA per murine GeCKO v2 half-library, which was a gift from Feng Zhang (Addgene plasmid # 1000000052), were amplified according to the protocol provided by the Zhang Lab and shared *via* Addgene (https://media.addgene.org/cms/filer_public/b5/fd/b5fde702-d02c-4873-806f-24ac28b2a15a/geckov20_library_amplification_protocol_1.pdf). The B half-library was amplified twice, the A half-library once. These three libraries were mixed, positive control sgRNAs targeting the reporters were spiked in to a concentration of about 0.07% and the resulting mix was used for lentivirus production. The screening cell line was infected at an MOI of about 0.5 and selected with Puromycin. Redundancy was kept at about 170 throughout each replicate of the screen. Six days after infection, cells were analyzed by FACS and sorted into lysis buffer. The same number of cells, which were sorted, were also lysed and analyzed as the “unsorted” control. Genomic DNA was extracted by phenol-chloroform extraction, the sgRNAs were amplified via two PCR reactions (3 μg genomic DNA or 1 ng GeCKO library was used for the first PCR; 1 μL of the first PCR reaction was used for the second PCR, which introduced the flow cell binding sequences and indices). The second PCR reactions were gel-purified and Illumina sequencing was performed. Reads were trimmed using cutadapt (v1.18) and aligned to sgRNA sequences using bowtie2 (v2.3.4) (Langmead and Salzberg, 2012) in stranded mode. Information about the sgRNAs was obtained from the annotations hosted on the MAGeCK sourceforge page, although these annotations were adapted to include the control sgRNAs only once and to include the positive control sgRNAs that were spiked in (Li et al., 2014). The reads were further sorted using samtools (v1.9) (Li et al., 2009) and were assigned to sgRNAs using the MAGeCK (v0.5.7) (Li et al., 2014) *count* command. Enrichment was calculated using the MAGeCK *test* command in positive selection mode and with “totalˮ as normalization strategy by using the command line arguments*--norm-method total--sort-criteria pos*.

### RNA-sequencing

About 48 h after siRNA transfection, NIH/3T3 cells were harvested, ERCC RNA (Invitrogen) was spiked in (for R1 in lysis buffer, for R2 and R3 after RNA extraction) and RNA was isolated using the RNeasy Mini Kit according to manufacturer’s instructions. Libraries were prepared using the NEBNext Poly (A) mRNA Magnetic Isolation Module and the NEBNext Ultra Directional RNA Library Prep Kit for Illumina according to manufacturer’s instructions. Sequencing was performed on the NextSeq 500 Sequencing System. Alignment to mm 10 was performed using bowtie2-2.2.7. Reads were randomly subsampled to equal numbers per sample. Count tables were created using Genomic Alignments in R. Genes with fewer than 12 reads across all samples were dropped. Differential expression analysis was performed using edgeR (Robinson et al., 2010).

### Pre-ranked gene set enrichment analysis

Count tables and gene expression analysis was performed as described in sections “RNA-Sequencing (RNA-Seq)” and “4-thiouridine sequencing (4sU-Seq)”. *p*-values were used for ranking according to the following formula: $I\left(\log FC\geq 0\right)\times -\log_{10}\left(p\right)$ with the indicator function I being 1 if true and -1 otherwise, i.e., the log10 of the *p*-value was calculated and the sign was used to indicate the direction of the logFC. The gene sets analyzed were downloaded from the MSigDB database: “GOBP_RIBOSOME_BIOGENESIS.v7.5.1”, “GOCC_ORGANELLAR_RIBOSOME.v7.5.1” and “GOCC_CYTOSOLIC_RIBOSOME.v7.5.1”.

### Knock-in cell line generation

The sgRNAs were designed manually in a way that the cut would be close to the start codon. Human U2OS cells were transfected using PEI and the sgRNA construct and a homology-repair template including the AID-tag, V5-tag and a Blasticidin resistance gene. Cells were selected and a single cell clone harboring the homozygous knock-in was generated.

### 4-Thiouridine sequencing

Cells were treated with 500 µM Indole-3-acetic acid (auxin) for 6 h and labelled with 4sU during the last 15 min of treatment. 4sU-labelled T cell lysates were spiked in. Cells were harvested in QIAzol and RNA was extracted using the miRNeasy mini kit according to manufacturer’s instructions. RNA was precipitated, labelled with biotin and pulled-down with Straptavidin-coated beads. Library was prepared using the NEBNext rRNA Depletion Kit according to manufacturer’s instructions. Sequencing was performed on the NextSeq 500 from Illumina. Reads were mapped to hg19 with Bowtie2 (reads mapping to rRNA, exons and blacklist regions were removed for the analysis, in which exonic reads were excluded). Count tables were created using GenomicAlignments in R. Differential expression was analyzed on genes with more than a sum of 90 counts over all conditions (and logCPM > 2 for the analysis, in which exonic reads were excluded). Normalization and differential expression analysis were performed using EdgeR (Robinson et al., 2010).

### Growth curve

A cumulative growth curve was performed on U2OS cells treated with 500 µM auxin. A standard deviation of the triplicates was calculated and the data were plotted with R.

### Fluorescence-activated Cell Sorting analysis general

In general, FACS analyses were performed by gating for living cells appearing within a cloud in a plot of SSC-A against FSC-A. Singlets were then gated in a plot showing FSC-W against FSC-H and SSC-W against SSC-H. Based on these events, usually 10.000 cells were analyzed per sample. Data were afterwards analyzed using FlowJo v10 and slightly different gatings: firstly, SSC-A vs. FSC-A, then FSC-H vs. FSC-A. A histogram view was used for the figures with a biexponential representation of the events on the x-axis and normalization to mode on the y-axis. Curves were smoothed. Statistics for the FACS analyses can be found in Supplementary Table S6.

### Annexin V/propidium iodide-Fluorescence-activated Cell Sorting

500 µM auxin was added for 48 h. Cells were harvested, labelled with Annexin V and propidium iodide (PI) and analyzed by FACS. Four gates (Annexin V-/PI-, Annexin V+/PI-, Annexin V-/PI+ and Annexin V+/PI+) were set and the percentage of early apoptotic (Annexin V+/PI- cells) and late apoptotic (Annexin V+/PI+) cells was plotted.

### Rescue experiment

The screening cell line was infected with lentiviral constructs (pLT3 backbone) that express the following shRNAs and proteins in a Doxycycline-inducible manner: 1) shRNA targeting *Renilla Luciferase* and overexpression of a non-fluorescent enhanced GFP variant (EGFP^R96H^) (control condition), 2) shRNA targeting *Aldoa* and overexpression of the non-fluorescent EGFP^R96H^ (sh*Aldoa* condition), 3) shRNA targeting *Aldoa* and overexpression of wild type ALDOA (“sh*Aldoa* + ALDOA^wt^” condition), 4) shRNA targeting *Aldoa* and overexpression of ALDOA^D34S^ (“sh*Aldoa* + ALDOA^D34S^” condition) or 5) shRNA targeting *Aldoa* and overexpression of ALDOA^K147Q^ (“sh*Aldoa* + ALDOA^K147Q^” condition). After infection, cells were selected with Puromycin for three days. 5 × 10^5^ cells were seeded in duplicates. The next day, cells were treated with doxycycline (Dox) (1 μg/ml) or with the vehicle ethanol (EtOH) for three days. The medium was exchanged daily. After three days, cells were trypsinized and kept in 2% FCS/PBS on ice until flow cytometry measurement. At least 1 × 10^4^ events were recorded for each condition. GFP and RFP were measured (FITC-A and PE-A). Uninfected NIH/3T3 cells were used as a negative control.

## Results

### Generation of a reporter cell line for ribosomal biogenesis gene expression

**FIGURE 1:**
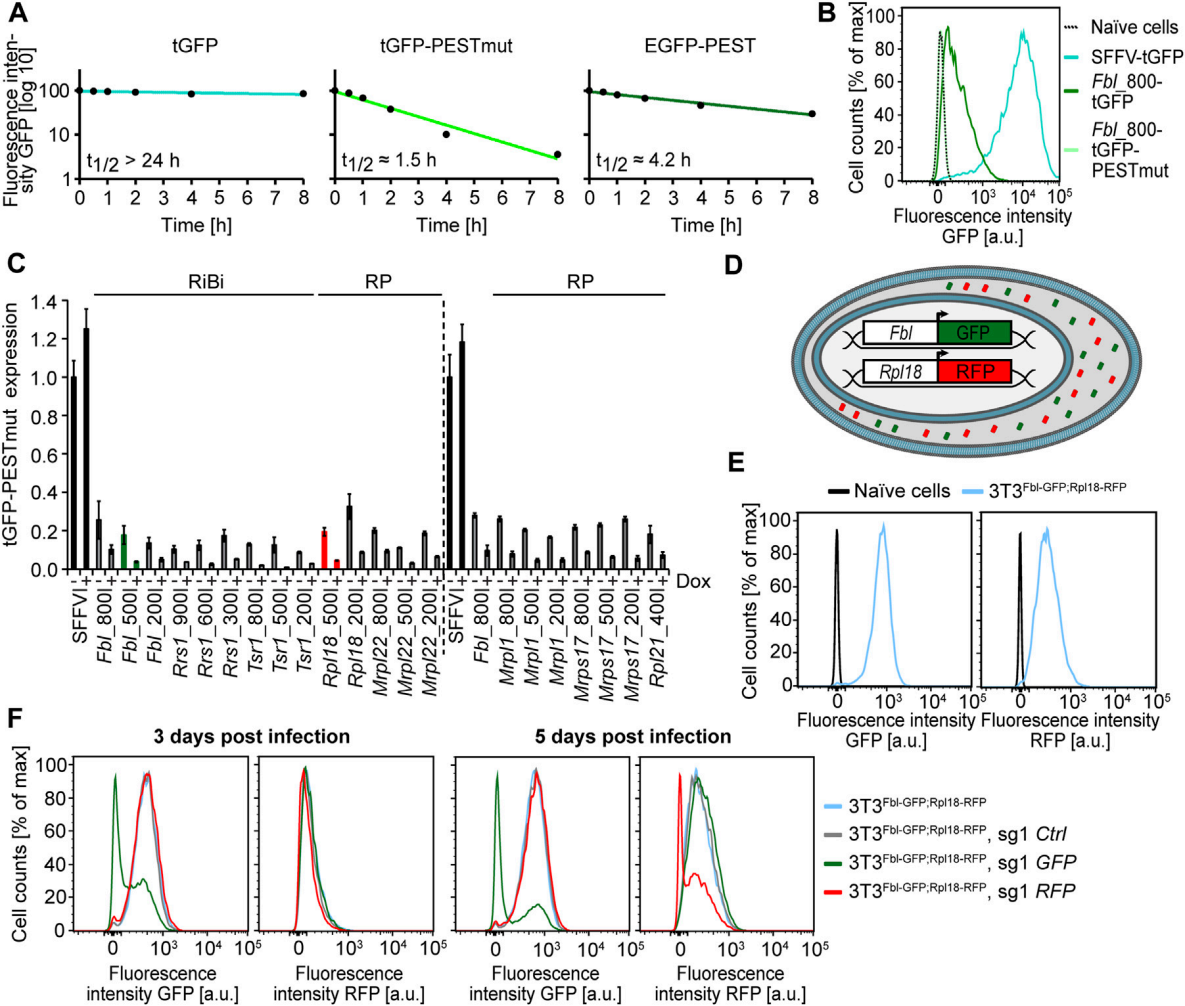
Reporter and promoter choice, generation of the screening cell line and testing of positive control sgRNAs. **(A)** Protein stability assay on different green fluorescent protein reporters. Median green fluorescence of TurboGFP (left panel), TurboGFP fused to a triple alanine mutated (E428A/E430A/E431A) PEST domain of the mouse ornithine decarboxylase (mODC) protein (middle panel), and enhanced GFP (eGFP) fused to the wild type mODC PEST domain (right panel), (Left panel) CHX (100 μg/ml) assay on HEK cells expressing SFFV-driven TurboGFP (tGFP) Single experiment, (Middle panel) CHX (100 μg/ml) assay on HEK cells expressing SFFV-driven tGFP-PESTmut Single experiment, (Right panel) CHX (10 μg/ml) assay on U2OS cells expressing SFFV-driven EGFP-PEST. Single experiment. **(B)** Fluorescence intensity of GFP variants driven by different reporters. Murine embryonic fibroblast (MEF) cells were infected with the indicated reporter constructs and analyzed by FACS. Single experiment. Naïve cells = uninfected MEFs, SFFV = spleen focus forming virus promoter, *Fbl_*800 = *Fibrillarin* promoter fragment of approx. 800 bp. **(C)** Quantitative real-time PCR (qRT-PCR) of RiBi- and RP-promoter-driven tGFP-PESTmut reporter transcripts upon *MYC* depletion. T lymphoma^MYC−Tet-Off^ cells were infected with the indicated reporter constructs, which are labelled to reflect the respective gene promoter and its length (in bp), and treated with Dox (1 μg/ml) for 16 h–24 h. Expression was analyzed normalized to *ß-Actin*. The dashed line separates two different runs. Representative experiment from a duplicate (for some promoters a triplicate) experiment. The green and red promoters were chosen for further usage in the screen. RiBi = Ribosome biogenesis gene promoter, RP = ribosomal protein gene promoter. Error bars represent standard deviation of technical triplicates. **(D)** Schematic representation of the cell line used for the genome-wide reporter screen. EGFP-PEST (GFP) expression is driven by the murine *Fibrillarin* (*Fbl*) promoter. tRFP-PEST (RFP) expression is driven by the murine *Ribosomal protein L18* (*Rpl18*) promoter. The EGFP-PEST and tRFP-PEST proteins are depicted in the cytoplasm as green and red barrels, respectively. **(E)** FACS analysis of the cell line used for the genome-wide reporter screen. 3T3^*Fbl*−GFP;*Rpl18*−RFP^ is a murine NIH/3T3 cell line, that expresses EGFP-PEST (GFP) under the control of the approx. 500 bp *Fbl* promoter fragment and tRFP-PEST (RFP) under the control of the approx. 500 bp *Rpl18* promoter fragment. Naïve NIH/3T3 = uninfected NIH/3T3 cells.**(F)** FACS analysis of the time-dependent effects of sgRNAs targeting EGFP-PEST (GFP) or tRFP-PEST (RFP) on reporter expression. Three days (left panel) or five days (right panel) after infection of the screening cell line 3T3^*Fbl*−GFP;*Rpl18*−RFP^ Single experiment.

To identify novel regulators of ribosomal biogenesis by genetic screens, we aimed to generate a stable cell line, in which fluorescent proteins indicate the expression of ribosomal biogenesis genes. Various versions of fluorescent proteins are available and the literature indicates a wide variety of spectral features and protein stabilities (Lambert, 2019). For genetic screens, it is important that the fluorescent reporter is robustly detectable, but at the same time not too stable, as the fluorescent signal should rapidly respond to changes in promoter activity caused by the genetic perturbation. We therefore constructed three green fluorescent proteins: (i) enhanced GFP (EGFP) fused to a degron of mouse ornithine decarboxylase (mODC) that contains a PEST motif (GFP-PEST), (ii) TurboGFP (tGFP) fused to the PEST motif containing 3 mutations (GFP-PESTmut), which are reported to further increase protein degradation (Li et al., 1998), and (iii) tGFP without any additional degron (GFP). We stably expressed all three GFP variants from a universal promoter (SFFV) in various human cell lines by lentiviral transduction. We then measured the half-life of all three fluorescent proteins by Fluorescence-activated Cell Sorting (FACS) upon incubation with the translation inhibitor cycloheximide for various time periods (Figure 1A). While untagged GFP is stable (t_1/2_ GFP > 24 h), the half-life drops clearly when GFP is expressed together with the PEST motif (t_1/2_ GFP-PEST: 4.2 h). The mutated PEST domain further decreases protein stability (t_1/2_ GFP-PESTmut: 1.5 h), but is almost undetectable by FACS and not suitable as a reporter in our cell line (Figure 1B). We therefore chose fluorescent proteins fused to the wild type PEST domain of mODC for the screen.

We next aimed to select promoters of genes involved in ribosomal biogenesis as reporters. We considered promoters of 5 murine ribosomal protein (RP) genes and 3 murine ribosomal biogenesis factors (RiBi). We cloned various different regions spanning between 200 and 900 nucleotides of each promoter upstream of the GFP reporter and stably expressed the constructs in a murine T lymphoma cell line. We chose this cell line, as it contains a doxycycline responsive allele of the MYC oncogene (Felsher and Bishop, 1999). MYC is a transcription factor and activates the expression of both RP and RiBi genes (Sabo et al., 2014; Lorenzin et al., 2016). qPCR analysis upon doxycycline treatment and concomitant MYC depletion revealed that *GFP* expression and regulation by MYC is different for the tested promoter fragments (Figure 1C). Based on these results, in conjunction with further FACS analyses, we selected the 564 nucleotides long *Fibrillarin* (*Fbl*) promoter and the 569 nucleotides long promoter of the *Large Ribosomal Subunit Protein 18* (*Rpl18*) as suitable promoters for the phenotypic screen.

In order to generate a cell line, in which both, RP and RiBi expression can be estimated, we cloned these promoters upstream of the selected fluorescent protein reporters (*Fbl*-GFP, *Rpl18*-RFP), respectively (Figure 1D). We then transduced murine fibroblast (NIH/3T3) cells with both constructs and selected a single cell clone (3T3^*Fbl*−GFP;*Rpl18*−RFP^), which shows high and comparable expression of both reporters (Figure 1E). We aimed to identify novel regulators of ribosomal biogenesis by a CRISPR-screen in this cell line and thus firstly analyzed how fast reporter activity decreased after the expression of single guide (sg) RNAs targeting *GFP*, *RFP* or a non-targeting control. We therefore transduced 3T3^*Fbl*−GFP;*Rpl18*−RFP^ cells with the respective sgRNAs and analyzed fluorescence 3 and 5 days after infection. Both, RFP and GFP expression was strongly reduced when treated with the corresponding, but not the other sgRNAs (Figure 1F; Supplementary Table S6). However, GFP expression was already partly reduced after 3 days while RFP seems unaffected at this earlier time point, suggesting that RFP is more stable than GFP although both proteins contain the same PEST sequence. We concluded that 3T3^*Fbl*−GFP;*Rpl18*−RFP^ is a suitable cell line for CRIPSR-based genetic screens and that reporter levels can be analyzed already 5 days after sgRNA transduction.

### Genome-wide screen for novel regulators of ribosomal biogenesis

**FIGURE 2:**
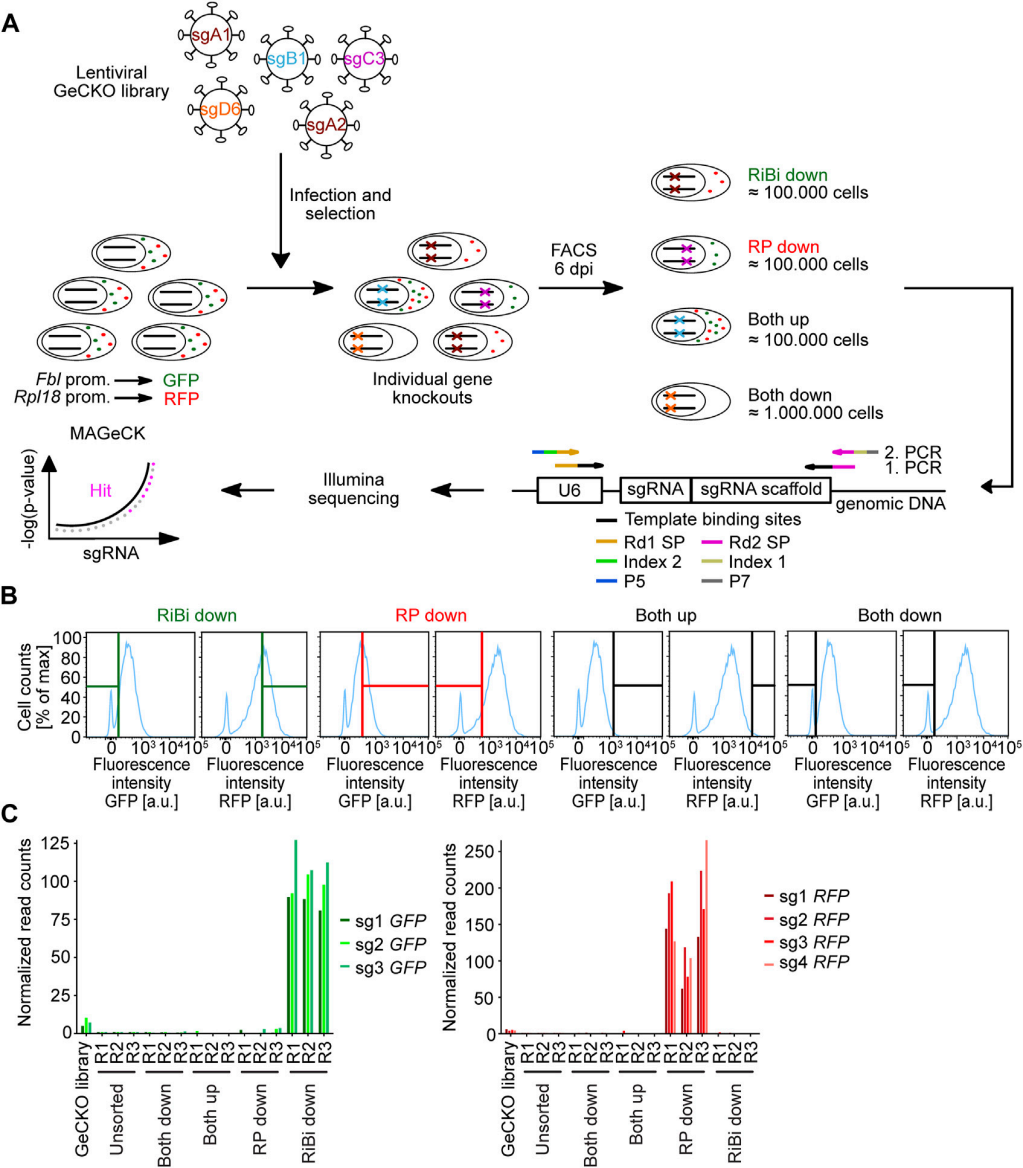
Screening procedure and enrichment of positive control sgRNAs across the different conditions. **(A)** Scheme of the genome-wide CRISPR/Cas9 screen in the 3T3^*Fbl*-GFP;*Rpl18*-RFP^ cell line. 3T3^*Fbl*−GFP;*Rpl18*−RFP^ is a murine NIH/3T3 cell line, that expresses EGFP-PEST (GFP) under the control of the approx. 500 bp *Fbl* promoter fragment and tRFP-PEST (RFP) under the control of the approx. 500 bp *Rpl18* promoter fragment. Prom. = promoter, dpi = days post infection, RiBi = ribosome biogenesis, RP = ribosomal protein, U6 = U6 promoter, sgRNA = single guide RNA, Rd1 SP or Rd2 SP = read sequencing primer, P5 and P7 = flow cell binding sites, MAGeCK = model-based analysis of genome-wide CRISPR/Cas9 knockout algorithm. **(B)** Gate settings used for sorting the screening samples. The cells were sorted for the indicated populations: cells that displayed high red fluorescence, but low green fluorescence, were sorted as the “RiBi downˮ fraction. Cells that showed high green fluorescence, but low red fluorescence, were sorted as the “RP downˮ fraction. Cells that presented high or low green and red fluorescence were sorted into the “Both upˮ or “Both downˮ" fractions, respectively. **(C)** Enrichment of positive control sgRNAs across the different screening conditions. Analysis of normalized read counts of sgRNAs targeting GFP or RFP in the different conditions of the screen as a ratio over normalized read counts of the unsorted condition.

We aimed to identify so far unknown regulators of ribosomal biogenesis by identifying genes that affect reporter activity in 3T3^*Fbl*−GFP;*Rpl18*−RFP^ in a genome-wide CRISPR screen (Figure 2A). We chose the publicly available GeCKO v2 plasmid library, which targets 20,611 murine genes with 6 sgRNAs per gene and contains 1,000 non-targeting sgRNAs (Sanjana et al., 2014). In total, it consists of 130,209 sgRNAs in two plasmid half-libraries of similar size. We first separately amplified both half-libraries in *E. coli* and then performed Illumina-sequencing of the variable guide region in order two estimate the abundance of the individual sgRNAs in the library pool. Global density plotting of the sgRNAs in both half-libraries demonstrate a narrow and similar distribution of all contained sgRNAs (Supplementary Figure S1A). The amplified half-libraries were then pooled and all steps of the screen were performed with sufficient cells (≈170-fold redundancy) to maintain the sgRNA complexity throughout the screen.

We started the screen by preparing pooled lentiviruses and transduced 3T3^*Fbl*−GFP;*Rpl18*−RFP^ cells. After selecting infected cells with Puromycin, we sorted cells for fluorescence reporter expression (Figure 2B). From the cell population, we selected cells with low GFP intensity indicating reduced activity of the *Fbl* promoter (“RiBi down”) or reduced RFP intensity indicating reduced *Rpl18* promoter activity (“RP down”). Hence, for both conditions, gates were chosen to select cells in which only one fluorescence reporter was downregulated. Additionally, we sorted cells which show reduced (“Both down”) or increased (“Both up”) fluorescence of both reporters (Figure 2B). The same number of cells used for sorting, was also collected (“unsorted”) in order to calculate enrichment/depletion of individual sgRNAs of the sorted conditions over this unsorted condition. We then isolated genomic DNA from all conditions and prepared indexed Illumina sequencing libraries by two consecutive PCR reactions (Supplementary Figures S1B, C).

Analysis of normalized read counts demonstrated a strong enrichment of sgRNAs targeting GFP or RFP in the respective conditions, indicating that FACS selected for cells with the correct fluorescence phenotype (Figure 2C).

**FIGURE 3:**
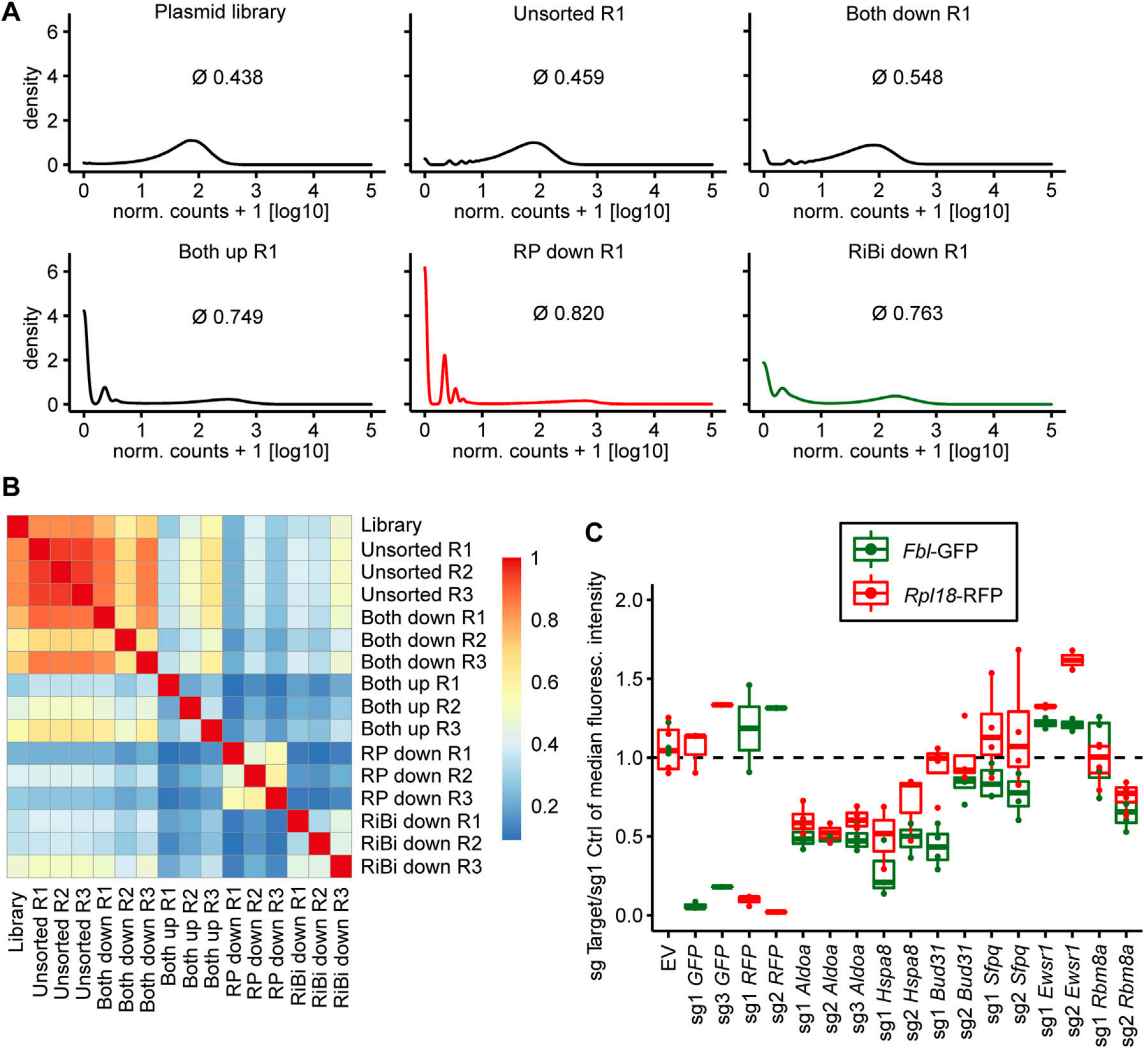
Quality assessment of the screen and validation experiments. **(A)** Kernel density estimate plots of normalized read count frequencies. Numbers indicate gini coefficient (mean over the three replicates). **(B)** Pearson’s correlation of the normalized read counts for each sample of the screen. **(C)** Boxplot depicting changes in fluorescence intensity of the screening cell line 3T3^*Fbl*-GFP;*Rpl18*-RFP^ upon candidate gene knockout. Background-subtracted median fluorescence intensity of the target sgRNA over a non-targeting control sgRNA is plotted. Boxes indicate 25th and 75th percentile. Horizontal lines within boxes indicate the median. Whiskers extend to 5th and 95th percentile. Dots represent individual experiments.

After completion of the genetic screen, we first analyzed its quality by estimating the global distribution of sgRNA in the various conditions by density plotting and calculating a Gini index, that is 0 for perfect homogeneity (all sgRNAs are represented by reads to the same degree) and 1 for maximal heterogeneity (all reads are from one sgRNA). We found the sgRNA distribution in unsorted cells to be as homogenous as in the initial plasmid pool (Figure 3A; 0.438 for the plasmid library, 0.459 for the “unsorted” condition), indicating that the production of viral particles and cellular transduction did not reduce sgRNA representation in the library. A similarly broad sgRNA distribution was observed in the “Both down” condition, indicating that no specific sgRNAs got enriched in cells with overall reduced reporter activity (0.548). In contrast, the sgRNA distribution was much more heterogenous in the “RP down” (0.820), “RiBi down” (0.763) and “Both up condition” (0.749), indicating that specific sgRNAs changed reporter activity and consequently got enriched by FACS (Figure 3A). Furthermore, we generally observed good correlation of the sgRNA distribution in the three replicates of each condition (Figure 3B). We concluded that the replicates of the screen led to reproducible results.

We compared the abundance of all sgRNAs at gene level in the sorted conditions as compared to unsorted cells (Figure 2A; Table 1-4) by utilizing the MAGeCK algorithm (Li et al., 2014). In agreement with the global statistical analyses, we could not identify any enriched gene, in the “Both down” condition (Table 1, Supplementary Table S7). However, in the “Both Up” condition, we identified sgRNAs of various subunits of the proteasome significantly enriched (Table 2, Supplementary Table S8). We believe it is likely that the fluorescence intensity in these cases increased post-transcriptionally and not by changes in reporter activity. While the “Both up” condition validates the enrichment of the correct cell populations by FACS in our screen, it did not yield in the discovery of novel repressors of ribosomal biogenesis. The “RP down” condition revealed significant enrichment of sgRNAs targeting RFP, which served as positive controls, and of sgRNAs targeting *Adrm1*, a proteasomal ubiquitin receptor (Table 3, Supplementary Table S9). Similar to the proteasomal subunits, which were enriched in the “Both up” condition, we believe, that fluorescence intensities of the reporters were probably regulated post-transcriptionally upon *Adrm1* knockout. In contrast, sgRNAs targeting several transcription-related genes were enriched in the “RiBi down” condition, indicating that they scored, because their knockout caused a reduction in reporter transcription (Table 4, Supplementary Table S10), for example sgRNAs targeting *Ssrp1* (a member of the FACT chromatin remodeling complex), *Cpsf3l* (a member of the Integrator complex), or *Gpn1* (a GTPase involved in the import of the two largest subunits of RNA Polymerase II into the nucleus) were enriched in this condition. Further, several subunits of the RNA Polymerase II (RNAPII), such as *Polr2l*, *Polr2e*, *Polr2c* and *Polr2h* were among the significantly enriched genes. Since many transcription-related genes were enriched, we focused on hits scoring in this “RiBi down” condition for the rest of the study.

**TABLE 1:** Genes enriched in Both Down.

Rank	Gene	Score	*p*-value	FDR
1	Recql4	0.000015111	0.000091265	0.909674
2	Cdh13	0.000024294	0.00014272	0.909674
3	Lrp5	0.000031048	0.00018187	0.909674
4	Senp1	0.000070412	0.00039651	0.909674
5	4930432M17Rik	0.000072879	0.00041058	0.909674
6	Itga11	0.000089514	0.0005047	0.909674
7	Olfr585	0.00009233	0.00051922	0.909674
8	Uqcrc1	0.00010309	0.00058915	0.909674
9	Olfr186	0.00011387	0.00064765	0.909674
10	Fgf1	0.00011716	0.00066304	0.909674

**TABLE 2:** Genes enriched in Both Up.

Rank	Gene	Score	*p*-value	FDR
1	Psmd6	3.7935E-11	2.1991E-07	0.000825
2	Psmc5	3.3373E-10	2.1991E-07	0.000825
3	Psmd11	1.1244E-09	2.1991E-07	0.000825
4	Psmc4	7.2005E-09	2.1991E-07	0.000825
5	Pabpn1	5.0741E-08	2.1991E-07	0.000825
6	Psmb4	1.6917E-07	2.1991E-07	0.000825
7	Psmb1	1.3146E-06	6.8173E-06	0.021924
8	Psmb7	1.6992E-06	9.0165E-06	0.025371
9	Psmd3	1.9537E-06	0.000010776	0.026953
10	Sap18	2.6345E-06	0.000012095	0.027228
11	Zcchc11	3.3571E-06	0.000016494	0.033753
12	Psma4	3.9824E-06	0.000019133	0.034653
13	Ewsr1	4.4745E-06	0.000020012	0.034653
14	Psmb6	5.1234E-06	0.000029249	0.04703

**Table 3:** Genes enriched in RP Down.

Rank	Gene	Score	*p*-value	FDR
1	tRFP	6.4987E-19	2.1991E-07	0.002475
2	Adrm1	3.5253E-12	2.1991E-07	0.002475
3	Depdc1a	5.4942E-06	0.000029688	0.222772
4	Vhl	0.00002206	0.000087306	0.491337
5	H60b	0.000038865	0.00016823	0.757426
6	Olfr799	0.00008618	0.00032349	0.948588
7	Pygb	0.000089456	0.00042509	0.948588
8	E130012A19Rik	0.000091852	0.00043389	0.948588
9	Vmn2r30	0.000097601	0.0003688	0.948588
10	A230065H16Rik	0.00013588	0.00052318	0.948588

**Table 4:** Genes enriched in RiBi Down.

Rank	Gene	Score	*p*-value	FDR
1	Aldoa	1.1915E-15	2.1991E-07	0.000381
2	EGFP	8.3331E-15	2.1991E-07	0.000381
3	Hspa8	2.3324E-13	2.1991E-07	0.000381
4	Bud31	3.0021E-12	2.1991E-07	0.000381
5	Polr2l	1.3724E-09	2.1991E-07	0.000381
6	Polr2e	2.7794E-09	2.1991E-07	0.000381
7	Cdc16	5.3516E-09	2.1991E-07	0.000381
8	Eif4a3	1.4423E-08	2.1991E-07	0.000381
9	Polr2c	2.2839E-08	2.1991E-07	0.000381
10	Polr2h	6.2568E-08	2.1991E-07	0.000381
11	Anapc1	8.8244E-08	2.1991E-07	0.000381
12	Eif4a1	1.6418E-07	2.1991E-07	0.000381
13	Gm21637	2.1266E-07	2.1991E-07	0.000381
14	Sfpq	5.445E-07	1.9792E-06	0.003182
15	Rpa1	1.2323E-06	6.8173E-06	0.01021
16	Plk1	1.4059E-06	7.2572E-06	0.01021
17	Itgav	2.5937E-06	0.000011655	0.015434
18	Cpsf3l	2.7927E-06	0.000013855	0.017327
19	Ewsr1	3.7882E-06	0.000017373	0.019566
20	Uba1	3.7922E-06	0.000018253	0.019566
21	Krt19	3.9152E-06	0.000018253	0.019566
22	Ssrp1	4.6219E-06	0.00002529	0.025878
23	Rbm8a	4.9424E-06	0.000028369	0.026609
24	Gpn1	6.3704E-06	0.000027929	0.026609
25	Rbm22	7.2356E-06	0.000044643	0.040198

To validate candidates identified by the genome-wide reporter screen, we first cloned individual sgRNAs for selected candidates (Figure 3C). The selection was based on the following considerations: we excluded sgRNAs targeting RNAPII core units, Cpsf3l or Gpn1, because of their general role in transcription. Additionally, we also checked, whether the proteins encoded by the candidate genes are known to localize to the nucleus, in order to increase the chances to find direct transcriptional regulators of ribosome biogenesis. Furthermore, we noted that several genes whose gene products are members of the anaphase-promoting complex (APC/C), an E3 ubiquitin ligase complex, were enriched at the top of our hit list. Examples are *Cdc16* (rank 7), *Anapc1* (rank 11), *Cdc20* (rank 26), but also others just a few ranks behind. We believed that it would be less likely, that this protein complex has an additional, yet unknown role in directly regulating transcription of genes involved in ribosome biogenesis, than that its canonical function might have influenced reporter activity indirectly, for example by inducing cell cycle arrest in M-phase, which might have led to a decrease in reporter expression, since transcription is basically shut down in M-phase cells. We then decided to further investigate six candidate genes (Figure 3C). We could validate the reduction in GFP fluorescence driven by the fibrillarin promoter in the case of *Aldoa* (3/3 sgRNAs), *Hspa8* (2/2 sgRNAs), *Bud31* (2/2 sgRNAs), *Sfpq* (2/2 sgRNAs) and *Rbm8a* (1/2 sgRNAs) depletion. In contrast, we did not observe a reduction in GFP reporter activity upon depletion of Ewsr1. Interestingly, in many cases, we also observed a reduction in RFP expression, which was driven by the *Rpl18* promoter. We hypothesized, that the identified factors might regulate both, *Fibrillarin* and Rpl18 transcription, and that they might have been identified exclusively in the “RiBi down” condition, because the GFP protein is less stable than RFP. We therefore generated a cell line in which we interchanged the fluorescent reporter proteins and respective promoters (“color switch control”). Although GFP in this cell line is driven by the *Rpl18* and not the *Fibrillarin* promoter (as in the cell clone screened with), most candidate sgRNAs still reduced GFP expression more than RFP expression (Supplementary Figure S2A). We concluded that the genetic screen identified new regulators of ribosomal biogenesis and ribosomal protein genes but could not identify specific regulators of one class of them.

### The glycolytic enzyme ALDOA regulates ribosomal biogenesis genes

**FIGURE 4:**
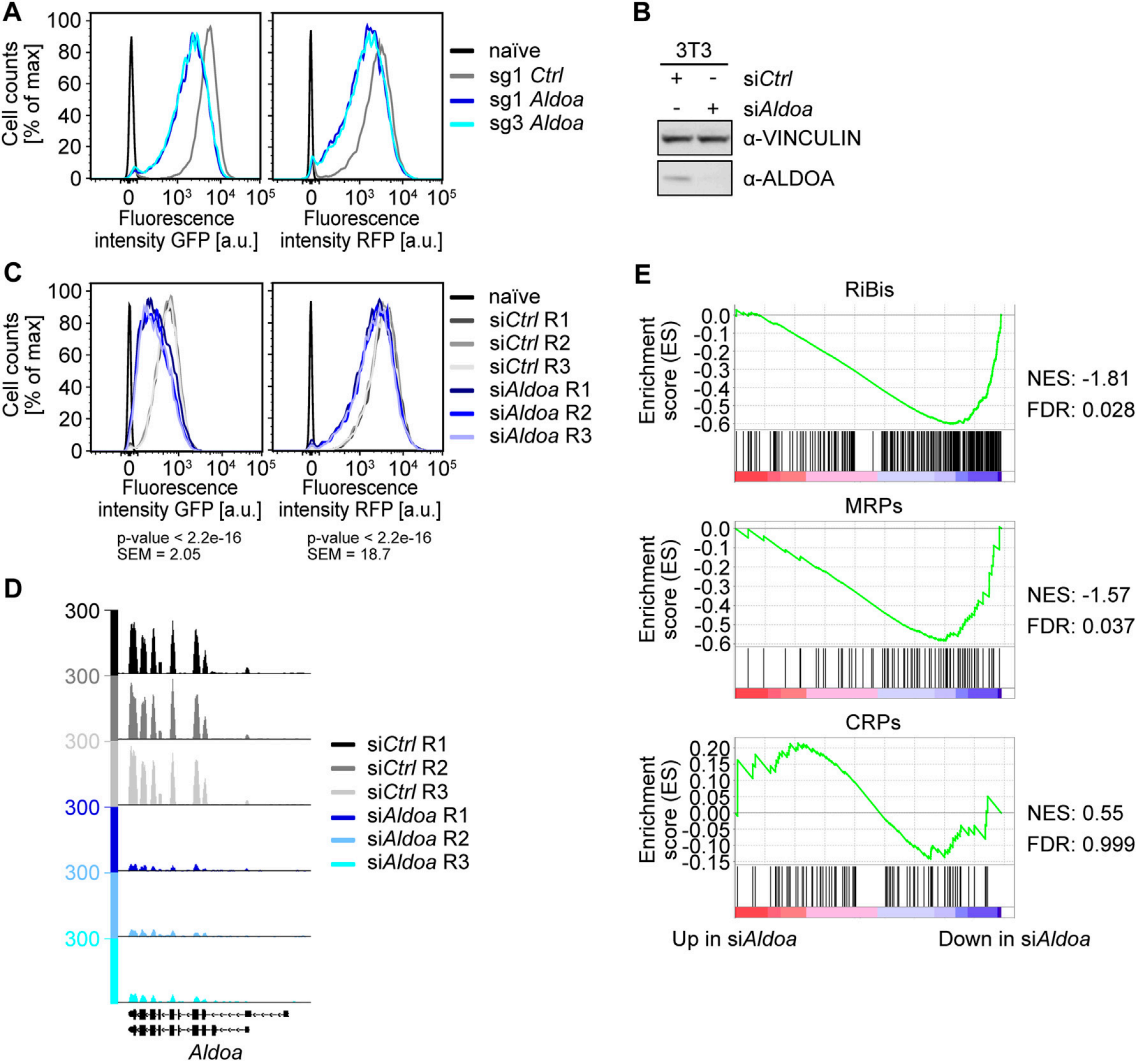
Analysis of the role of ALDOA in ribosome biogenesis. **(A)** FACS analysis of *Fbl*-promoter-driven EGFP-PEST (GFP) and *Rpl18*-promoter-driven tRFP-PEST (RFP) upon *Aldoa* knockout. FACS analysis of the screening cell line 3T3^*Fbl*−GFP;*Rpl18*−RFP^ six days after infection with sgRNAs targeting *Aldoa*. sg1 Ctrl = non-targeting control, naïve = wild type NIH/3T3 cells. A representative experiment from quadruplicate experiments is shown. **(B)** Western blot of ALDOA upon si*Aldoa* treatment. Cells were harvested 2 days after transfection of a pool of four siRNAs targeting *Aldoa* in NIH/3T3 cells. VINCULIN was used as a loading control. **(C)** FACS analysis of *Fbl*-promoter-driven EGFP-PEST (GFP) and *Rpl18*-promoter-driven tRFP-PEST (RFP) upon *Aldoa* knockdown. R1, R2, and R3 represent the different replicates. FACS analysis was performed three days after siRNA transfection. Mean fluorescence values (scale values) of the FITC-A channel for cells treated with si*Ctrl* or si*Aldoa* (triplicates were pooled for the analysis) were compared using a two-sided, unpaired Student *t*-test. Naïve = wild type NIH/3T3 cells, SEM = standard error of the mean. **(D)** Browser track picture of normalized *Aldoa* read counts. Cells were harvested 2 days after transfection of a pool of four siRNAs targeting *Aldoa* in NIH/3T3 cells. **(E)** Gene set enrichment analysis (GSEA) of ribosome biogenesis genes and ribosomal protein genes upon *Aldoa* knockdown. Genes were ranked by log10 (*p*-value) with the sign indicating the direction of the fold change (a negative fold change indicating downregulation upon *Aldoa* knockdown). The gene sets analyzed were downloaded from the MSigDB database: “GOBP_RIBOSOME_BIOGENESIS.v7.5.1”, “GOCC_ORGANELLAR_RIBOSOME.v7.5.1”, and “GOCC_CYTOSOLIC_RIBOSOME.v7.5.1”. RiBis = ribosome biogenesis genes, MRPs = mitochondrial ribosomal protein genes, CRPs = cytosolic ribosomal protein genes, NES = normalized enrichment score, FDR = false discovery rate.

Our genetic screen identified the gene encoding the glycolytic enzyme ALDOA as the highest scoring hit. We therefore focused on this protein and further analyzed if it regulates the expression of ribosomal biogenesis genes. After validating the effect of two *Aldoa*-targeting sgRNAs from the GeCKO v2 library on fluorescent reporter protein expression (Figure 4A, Supplementary Table S6), we analyzed if this effect can also be observed by using an independent depletion method to exclude off-target effects. We therefore transfected 3T3^*Fbl*−GFP;*Rpl18*−RFP^ cells with an siRNA targeting *Aldoa* or with a non-targeting control siRNA (Figure 4B). Gene silencing of *Aldoa* induced a substantial reduction in GFP and to a lesser extent RFP fluorescence (Figure 4C, Supplementary Table S6).

We then wondered, if depletion of *Aldoa* also induces transcriptional downregulation of endogenous ribosomal biogenesis genes. We therefore isolated RNA from siRNA-treated cells after 48 h and estimated mRNA levels by RNA-sequencing. The analysis of reads mapping to the *Aldoa* locus confirmed robust gene silencing in all three biological replicates (Figure 4D). Strikingly, gene set enrichment analysis revealed the significant downregulation of ribosome biogenesis genes (Figure 4E). Many mitochondrial ribosomal genes (MRP) were also downregulated upon *Aldoa* depletion, while genes of the cytosolic ribosome were not repressed in the absence of ALDOA (Figure 4E). Among the most repressed RiBi and MRP genes were *Ybey* (logFC −0.83, adj. *p*-value 0.006), *Mettl18* (logFC −0.75, adj. *p*-value 0.061), and *Mrpl55* (logFC −0.75, adj. *p*-value 0.001). We concluded that ALDOA activates the expression of endogenous ribosomal biogenesis genes in fibroblasts.

We wondered whether the glycolytic activity of ALDOA was required for the observed effect on ribosome biogenesis. Therefore, we made use of our reporter cell line and performed a rescue experiment with overexpression of wild type ALDOA or catalytically defective mutants (D34S, K147Q) (Morris and Tolan, 1993; Morris et al., 1996) upon shRNA-mediated knockdown of endogenous *Aldoa*. Only wild type ALDOA, but none of the mutants could rescue the shRNA-mediated decrease in reporter activity (Supplementary Figures S2B,C; Supplementary Table S6), suggesting that the glycolytic activity of ALDOA is needed for expression of ribosome biogenesis genes.

### The exon junction complex protein RBM8A regulates ribosomal protein genes

**FIGURE 5:**
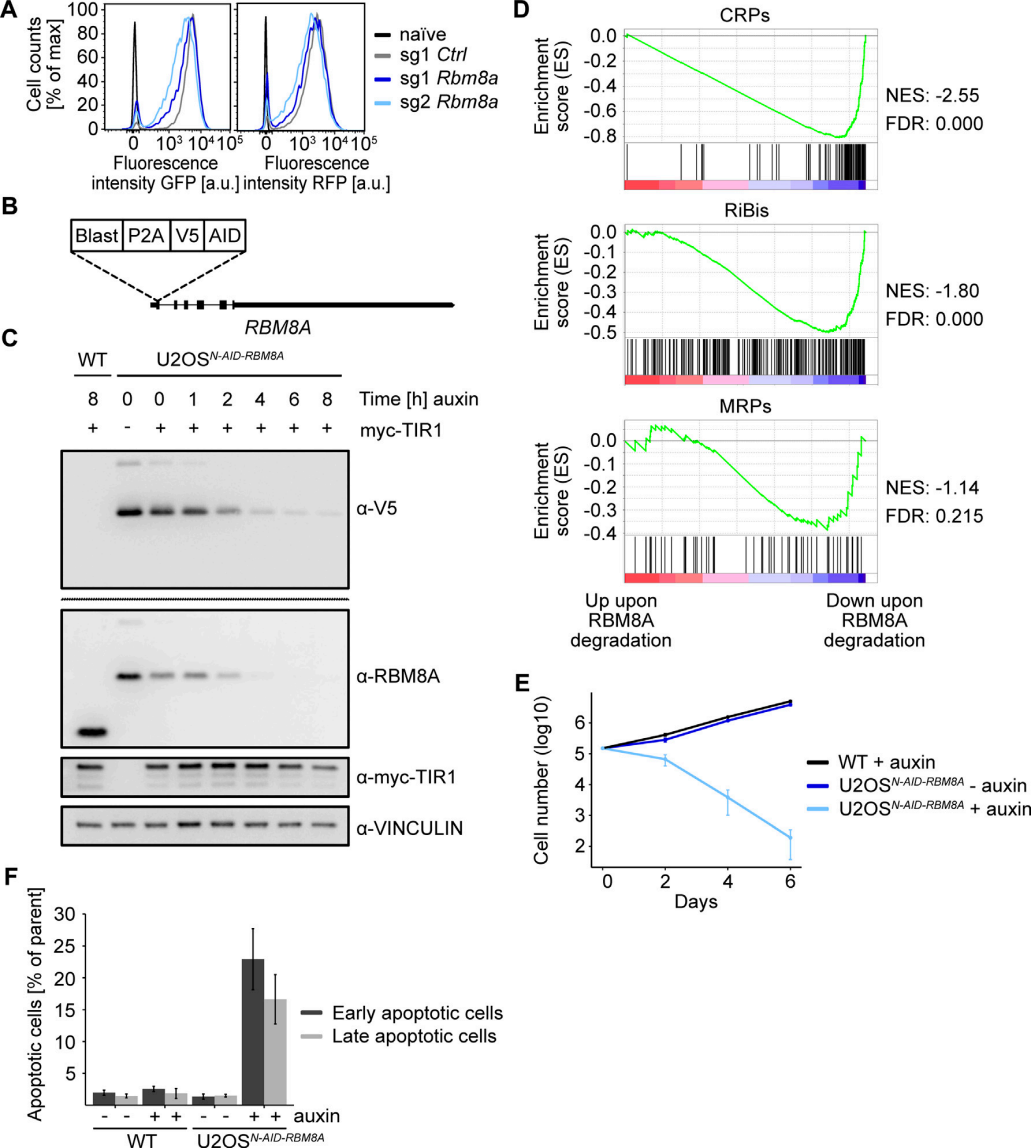
Analysis of the role of RBM8A in ribosome biogenesis. **(A)** FACS analysis of *Fbl*-promoter-driven EGFP-PEST (GFP) and *Rpl18*-promoter-driven tRFP-PEST (RFP) upon *Rbm8a* knockout. FACS analysis of two different sgRNAs targeting Rbm8a six days after lentiviral infection and selection of the screening cell line 3T3^Fbl−GFP;Rpl18−RFP^. sg1 *Ctrl* = non-targeting, naïve = wild type NIH/3T3 cells. Representative experiment from a quadruplicate experiment. **(B)** Scheme of the homozygous *N-AID-RBM8A* knock-in. Blast = Blasticidin, P2A = porcine teschovirus-1 2A self-cleaving peptide, V5 = V5-tag, AID = auxin-inducible degron tag. **(C)** Western blot of N-AID-RBM8A upon addition of auxin for different time periods. The U2OS^*N−AID-RBM8A*^cell clone was infected with virus encoding the F-box transport inhibitor response 1 (TIR1) protein and treated with 500 µM auxin for different time periods. The V5-blot was a whole membrane blot. Single experiment. WT = wild type U2OS cells. **(D)** Gene set enrichment analysis (GSEA) of ribosome biogenesis genes and ribosomal protein genes upon acute (6 h) RBM8A depletion and nascent RNA sequencing (4sU-Seq). Genes were ranked by log10 (*p*-value) with the sign indicating the direction of the fold change in the exon-excluding 4sU-Seq data (a negative fold change indicates downregulation upon RBM8A knockdown compared to the cell line without auxin treatment). The gene sets analyzed were downloaded from the MSigDB database: “GOBP_RIBOSOME_BIOGENESIS.v7.5.1”, “GOCC_ORGANELLAR_RIBOSOME.v7.5.1”, and “GOCC_CYTOSOLIC_RIBOSOME.v7.5.1”. RiBis = ribosome biogenesis genes, MRPs = mitochondrial ribosomal protein genes, CRPs = cytosolic ribosomal protein genes, NES = normalized enrichment score, FDR = false discovery rate. **(E)** Logarithmic (log10) growth curve of wild type (WT) TIR1-expressing U2OS cells treated with auxin and the U2OS^*N−AID-RBM8A*^cell clone ± auxin in triplicates. Error bars represent standard deviation of triplicates. **(F)** Annexin V/PI-FACS of WT TIR1-expressing U2OS cells and the U2OS^*N−AID-RBM8A*^cell clone±auxin in triplicates. 48 h auxin treatment. Early apoptotic cells are cells with high Annexin V, but low propidium iodide (PI) signal. Late apoptotic cells are cells with high Annexin V and high propidium iodide (PI) signal. Error bars represent standard deviation of triplicates.

Although the reporter genes used in this screen do not contain an intron, the exon junction complex protein gene *Rbm8a* scored in the genetic screen and we could validate the effect on reporter expression with one sgRNA targeting *Rbm8a* (Figure 5A, Supplementary Table S6). We therefore wondered if RBM8A also regulates the expression of endogenous ribosomal biogenesis genes. Since other groups reported the direct involvement of exon junction complex proteins in transcriptional regulation (Akhtar et al., 2019), we aimed to acutely deplete RBM8A and analyze nascent transcription. For rapid depletion, we used the inducible auxin degron system (Nishimura et al., 2009). U2OS cells were transiently transfected with an sgRNA targeting the *RBM8A* gene close to the start codon together with a repair DNA template, containing the auxin inducible degron (AID) tag and a V5 tag for unequivocal fusion protein detection and a Blasticidin selection cassette (Figure 5B). Transfected cells were selected with Blasticidin and genomic DNA was used to identify individual cell clones with a homozygous integration of the AID tag by PCR (Supplementary Figure S3A). We then sequenced the *RBM8A* gene to confirm correct integration of the AID cassette (Supplementary Figure S3B). Immunoblot analysis confirmed the AID-mediated shift of RBM8A and the detection of the V5 tag in the knock-in clone (U2OS^AID−RBM8A^, Figure 5C).

To analyze if and how transcription is directly regulated by RBM8A, we incubated U2OS^AID−RBM8A^cells with auxin for 6 h and then added 4-thiouridine (4sU), which is incorporated into nascent transcripts, for 15 min. Afterwards, 4sU-labeld transcripts were biotinylated, isolated and subjected to Illumina sequencing. Strikingly, GSEA analysis of the RiBi (ribosome biogenesis), CRP (cytosolic ribosomal protein) and MRP (mitochondrial ribosomal protein) gene sets revealed a significant downregulation of nascent transcript levels of mainly cytosolic ribosomal protein genes (Figure 5D). Since imbalances in ribosomal biogenesis can cause cell growth arrest and death, we characterized the cellular phenotypes upon sustained depletion of RBM8A. Incubation of U2OS^AID−RBM8A^cells with auxin dramatically decreased the cell number over 6 days, while untreated U2OS^AID−RBM8A^cells or wild type cells treated with auxin continuously proliferated (Figure 5E). Finally, we observed that RBM8A depletion causes rampant apoptosis after 48 h as measured by Annexin-positive cells using flow cytometry (Figure 5F). We concluded that RBM8A directly activates the transcription of ribosomal protein genes and is required for cell growth and proliferation.

We did not expect the exon junction complex protein *Rbm8a* (Le Hir et al., 2000) to regulate the activity of the intronless reporter used here. We therefore wondered whether intronless genes are regulated upon acute RBM8A depletion. We noticed that 99 intronless genes (of a total of 759 intronless genes) were downregulated (adjusted *p*-values < 0.05), while only 24 were significantly upregulated upon acute RBM8A depletion. We then globally analyzed RBM8A-mediated gene regulation and intron content and observed a positive correlation (Pearson correlation coefficient: 0.616, 95% CI: 0.334—0.796, Supplementary Figure S3C): The fewer introns a gene has, the stronger it appears to be activated by RBM8A. Markedly, this effect was strongest in genes containing no introns at all (Supplementary Figure S3C). An exemplary activated intronless and intron-containing gene are shown (Supplementary Figures S3D,E).

## Discussion

Ribosome biogenesis involves the synthesis of ribosomal proteins and rRNA on the one hand, and the production of proteins needed for the assembly and maturation of ribosomes on the other hand (Rudra and Warner, 2004). Although many of the proteins implicated in ribosome biogenesis are already known, the underlying molecular mechanisms of their regulation are not yet fully understood in mammals. Here, we identified new transcriptional regulators of ribosome biogenesis in a genome-wide knockout reporter screen utilizing a cell line containing fluorescent reports for the expression of ribosomal biogenesis factors (RiBi) and ribosomal protein genes (RP). The most comprehensive set of candidate genes was coming from the condition, where cells were sorted for low expression of *Fibrillarin* promoter-driven GFP and we could validate the effect on reporter activity for most hits. We then showed that the exon junction complex protein RBM8A and the glycolytic enzyme ALDOA activate the transcription of endogenous genes involved in ribosomal biogenesis. Interestingly, *Rbm8a* was also slightly downregulated in *Aldoa* depleted cells (log_2_FC of −0.358; adjusted *p*-value of 0.002), indicating a possible interplay of these two novel regulators of ribosome biogenesis.

The ALDOA enzyme catalyzes the reversible conversion of d-fructose-1, 6-bisphosphate (F1, 6-BP) to D-glyceraldehyde-3-phosphate (G3P) and dihydroxyacetone phosphate (DHAP) in the cytoplasm. However, many glycolytic enzymes are also present in the nucleus and diverse “moonlighting” functions apart from their established metabolic roles were identified for many of them (Ronai, 1993; Boukouris et al., 2016; Yu and Li, 2017). Hexokinase 2, for example, translocates to the nucleus and regulates gene expression in response to external stimuli (de la Cera et al., 2002; Ahuatzi et al., 2004; Neary and Pastorino, 2010). Strikingly, several reports indicate that also a fraction of ALDOA is localized inside the nucleus dependent on nutrient availability and cell density and upon other stimuli (Mamczur et al., 2013). ALDOA was shown to interact with DNA (Ronai et al., 1992) and the DNA-binding activity of ALDOA does not correlate with its glycolytic activity (Ronai, 1993). It is therefore surprising and interesting that the regulation of ribosomal biogenesis genes found here is dependent on the catalytic activity of ALDOA. Our observation that ALDOA regulates POL II activity on genes important for ribosomal biogenesis is in line with other studies, reporting that ALDOA associates with the POL III complex and enhances the binding of POL III to its target genes (Ciesla et al., 2014). Together with our observations these insights make ALDOA interesting for further investigations on how it regulates the transcription of RiBis and RPs.

Besides *Aldoa*, *Rbm8a* (also known as Y14) was another hit obtained from the condition sorted for low *Fibrillarin*-driven fluorescence. It is part of the exon-junction complex (EJC), which is loaded about 24 bp upstream of exon-exon junctions on mRNAs and affects several fates of mRNAs downstream of transcription, like splicing, mRNA localization, translation efficiency, and nonsense-mediated decay (Le Hir et al., 2000). Strikingly, not only *Rbm8a* scored as a significant hit, but *Eif4a3*, a gene encoding another core member of the EJC, also scored significantly in our screen. The reason, why we pursued to study the EJC is the fact, that both our reporter as well as many genes regulated by RBM8A did not contain introns. We thus find it unlikely that the canonical function of the EJC as a regulator of nonsense mediated decay can explain its influence on reporter activity and the transcriptional regulation of ribosomal biogenesis. Instead, we believe it is more likely that RBM8A directly regulates transcription of the respective genes, since rapid depletion of this protein by the auxin degron system acutely affects the levels of the nascent transcripts of these genes.

In line with this conclusion of our work, several published observations suggested already a possible role of the EJC in transcriptional regulation. The EJC is known to bind to promoters (Choudhury et al., 2016; Akhtar et al., 2019) and to associate with POL II (Chuang et al., 2019). Furthermore, interaction of EJC components with POL II was shown to affect promoter-proximal pausing, with a knockdown of the EJC leading to POL II pause-release and premature elongation (Wang et al., 2014; Akhtar et al., 2019). While increased POL II pause-release upon EJC depletion could lead to an increase of reporter signal, we instead observed that reporter expression is decreased upon *Rbm8a* knockout. The AID knock-in cell line of *RBM8A* provides a perfect tool for the further mechanistic evaluation of RBM8A in transcription.

## Data Availability

The datasets presented in this study can be found in online repositories. The names of the repository/repositories and accession number(s) can be found below: Genome-wide screen: GSE207900, https://www.ncbi.nlm.nih.gov/geo/query/acc.cgi?acc=GSE207900. RNA-Seq: GSE207899, https://www.ncbi.nlm.nih.gov/geo/query/acc.cgi?acc=GSE207899. 4sU-Seq: GSE207898, https://www.ncbi.nlm.nih.gov/geo/query/acc.cgi?acc=GSE207898.

## Supplementary material

Supplementary Figure S1 GeCKO v2 library amplification and sgRNA library preparation from genomic DNA. **(A)** Distribution of the guide RNAs around the median of each individually amplified half-library from the murine GeCKO v2 library. The read count of each sgRNA was divided by the median count for each half-library and a pseudocount of 0.001 was added. A density plot of the log10 transformed data was plotted for the amplified half-libraries. The B half-library was amplified twice and mixed with the A half-library. The mix was used for the screen. **(B)** Agarose gel of the first PCR of the screening sgRNA library preparation from genomic DNA. PCR products of the first PCR performed to amplify the guides from genomic DNA extracted from the different sorted conditions and unsorted cells. # corresponds to 28 PCR cycles, ‡ corresponds to 32 cycles. 25 cycles were used to amplify the unsorted samples during the first PCR. R1, R2, R3 = biological replicates. **(C)** Agarose gel of the second PCR of the screening sgRNA library preparation from genomic DNA. PCR products of the second PCR, which was performed to introduce the flow cell binding sequences. R1, R2, R3 = biological replicates

Supplementary Figure S2 Hit validation of the genetic screen. **(A)** Boxplot depicting changes in fluorescence intensity of the swapped reporter cell line 3T3^Rpl18-GFP;^ ^Fbl-RFP^ upon candidate gene knockout. Background-subtracted median fluorescence intensity of the target sgRNA over a non-targeting control sgRNA is plotted. Horizontal lines within boxes indicate the median. Single experiments are shown. **(B)** FACS analysis of the expression of Fbl-promoter-driven EGFP-PEST (GFP) and Rpl18-promoter-driven tRFP-PEST (RFP) upon Aldoa depletion and rescue with the indicated constructs. FACS analysis of a rescue experiment performed 3 days after Doxycycline (1 μg/ml)-induced expression of an shRNA targeting Aldoa and concomitant expression of ALDOA^wt^ or ALDOA^D34S^ or the non-fluorescent EGFP^R96H^ variant (shAldoa condition) in the screening cell line 3T3^Fbl—GFP;Rpl18—RFP^. An shRNA targeting Renilla Luciferase and a concomitant overexpression of EGFP^R96H^ was used as a control condition. Naïve = wild type NIH/3T3 cells. Representative experiment from a duplicate experiment is shown. **(C)** FACS analysis of the expression of Fbl-promoter-driven EGFP-PEST (GFP) and Rpl18-promoter-driven tRFP-PEST (RFP) upon Aldoa depletion and rescue with the indicated constructs. FACS analysis of a rescue experiment performed 3 days after Doxycycline (1 μg/ml)-induced expression of an shRNA targeting Aldoa and concomitant expression of ALDOA^wt^ or ALDOA^K147Q^ or the non-fluorescent EGFP^R96H^ (shAldoa condition) in the screening cell line 3T^3Fbl—GFP;Rpl18—RFP^. An shRNA targeting Renilla Luciferase and a concomitant overexpression of EGFPR96H was used as a control condition. Naïve = wild type NIH/3T3 cells. Representative experiment from a duplicate experiment is shown

Supplementary Figure S3 Generation and analysis of U2OS^*N−AID-RBM8A*^ cells. **(A)** Agarose gel of genotyping PCR of the U2OS^*N−AID-RBM8A*^ knock-in cell line. Genotype was compared to WT U2OS cells. kb = kilobase pairs, WT = wild type. **(B)** Sanger sequencing of a genotyping PCR product of the U2OS^*N−AID-RBM8A*^ cell line. Prom = Promoter, 5′hom/3′hom = 5′ or 3′ homology arm, MluI/EcoRI = restriction sites, Kozak = Kozak sequence, Blast = Blasticidin, AID = auxin-inducible degron, hg38 = the human reference genome hg38, chr = chromosome. **(C)** Scatterplot of the fold change upon RBM8A depletion vs. the intron number. The median log_2_FC was plotted against the rounded median intron count per gene, which was calculated from data acquired from Ensembl BioMart. 4sU-Seq experiment as in Figure 5D, but exonic reads were retained in the analysis. **(D)** Browser track picture of an exemplary intronless gene downregulated upon acute (6 h) RBM8A depletion. Browser track of normalized read counts of replicate 2 of the 4sU-Seq analysis as used in Supplementary Figure S3C. **(E)** Browser track picture of an exemplary intron-containing gene downregulated upon acute (6 h) RBM8A depletion. Browser track of normalized read counts of replicate 2 of the 4sU-Seq analysis as used in Figure 5D.

Supplementary Table S1 List of antibodies used.

Supplementary Table S2 List of the amino acid sequences of the fluorescent reporters used.

Supplementary Table S3 List of oligonucleotides used.

Supplementary Table S4 Differential expression results of the 4sU-Seq analysis excluding exonic reads.

Supplementary Table S5 Differential expression results of the 4sU-Seq analysis containing intronic and exonic reads.

Supplementary Table S6 Statistics of the FACS analyses.

Supplementary Table S7 Positively enriched genes from the “Both down” condition of the genome-wide CRISPR-Cas9 knockout screen. The genes represent genes from the condition sorted for low GFP (Fbl promoter) and low RFP (Rpl18 promoter) fluorescence intensity. The ranked gene list was obtained using the bioinformatic tool MAGeCK (Li et al., 2014).

Supplementary Table S8 Positively enriched genes from the “Both up” condition of the genome-wide CRISPR-Cas9 knockout screen. The genes represent genes from the condition sorted for high GFP (Fbl promoter) and high RFP (Rpl18 promoter) fluorescence intensity. The ranked gene list was obtained using the bioinformatic tool MAGeCK (Li et al., 2014).

Supplementary Table S9 Positively enriched genes from the “RP down” condition of the genome-wide CRISPR-Cas9 knockout screen. The genes represent genes from the condition sorted for constant or high GFP (Fbl promoter), but low RFP (Rpl18 promoter) fluorescence intensity. The ranked gene list was obtained using the bioinformatic tool MAGeCK (Li et al., 2014).

Supplementary Table S10 Positively enriched genes from the “RiBi down” condition of the genome-wide CRISPR-Cas9 knockout screen. The genes represent genes from the condition sorted for constant or high RFP (Rpl18 promoter), but low GFP (Fbl promoter) fluorescence intensity. The ranked gene list was obtained using the bioinformatic tool MAGeCK (Li et al., 2014).
