# Erratum: The glycolytic enzyme ALDOA and the exon junction complex protein RBM8A are regulators of ribosomal biogenesis

**DOI:** 10.3389/fcell.2022.1069902

**Published:** 2022-10-28

**Authors:** Frontiers Production Office

**Affiliations:** Frontiers Media SA, Lausanne, Switzerland

**Keywords:** ribosome biogenesis, ribosomal protein gene, genetic screen, genome-wide screen, RBM8A, Y14, AldoA, aldolase A

Due to a production error, the supplementary images were erroneously omitted from the original published article’s Supplementary Materials.

The publisher apologizes for this error and states that this does not change the scientific conclusions of the article in any way. The original article has been updated.

